# Quantitative Assessment of Visual Function in Japanese Patients With Lecithin-Cholesterol Acyltransferase Gene Abnormalities: A Case-Control Study

**DOI:** 10.7759/cureus.99227

**Published:** 2025-12-14

**Authors:** Takashi Ono, Takuya Iwasaki, Toshihiro Sakisaka, Yosai Mori, Ryohei Nejima, Kazunori Miyata

**Affiliations:** 1 Ophthalmology, University of Tokyo Hospital, Tokyo, JPN; 2 Ophthalmology, Miyata Eye Hospital, Miyakonojo, JPN

**Keywords:** contrast sensitivity, corneal opacity, familial lecithin-cholesterol acyltransferase deficiency syndrome, fish eye disease, lcat deficiency, visual function

## Abstract

Introduction

Fish-eye disease (FED) and familial lecithin-cholesterol acyltransferase (LCAT) deficiency (FLD) are rare. The aim of this study was to compare visual function between patients with LCAT abnormalities - namely, FED and FLD - and healthy controls.

Methods

This retrospective, comparative case-control study included four patients with FLD or FED (LCAT group) who presented with cloudy corneas at Miyata Eye Hospital between 2018 and 2024. Four age- and sex-matched individuals with normal results on ophthalmic examination were included as controls. We reviewed medical records for best-corrected visual acuity (BCVA), corneal astigmatism, forward light scattering, and contrast sensitivity. The parameters were compared between the groups.

Results

Sixteen eyes of eight women were included, including eight eyes of four patients with cloudy corneas in the LCAT group (two with FLD and six with FED) and eight eyes of four controls. The mean BCVA and corneal astigmatism revealed no significant intergroup differences. However, forward scattering was significantly higher in the LCAT group than in the control group (p = 0.007). The area under the log-contrast sensitivity function was significantly lower in the LCAT group than in the control group (p = 0.017).

Conclusions

Despite normal BCVA, patients with LCAT abnormalities (FLD and FED) showed considerably increased forward light scattering and decreased contrast sensitivity compared with the controls, indicating subtle but substantial visual functional impairment.

## Introduction

Lecithin-cholesterol acyltransferase (LCAT) is critical for the maturation of high-density lipoprotein cholesterol [1,2]. LCAT abnormalities result in two rare autosomal recessive disorders: familial LCAT deficiency (FLD) and fish-eye disease (FED) [3]. FED is characterized by abnormalities only in the eye, whereas FLD is associated with renal dysfunction with poor prognosis, diagnosed in 33 racial groups worldwide [4]. Both conditions are characterized by low serum high-density lipoprotein levels and bilateral corneal opacification [5]. The eyes of patients with FED present considerable difficulty for ophthalmologists, because examination of the anterior chamber, iris, lens, vitreous, and retina is extremely challenging. Although these patients subjectively perceive significant visual impairment, their visual acuity under standard testing in bright environments does not generally show deterioration. However, this does not imply the absence of functional disability, since visual function other than visual acuity, such as contrast sensitivity, is reduced under dim light conditions [6]. Congenital conditions such as corneal dystrophy, and acquired conditions such as infection, trauma, surgery, and corneal opacities, decrease contrast sensitivity and increase forward light scattering, and changes in the corneal state, such as keratoconjunctivitis sicca, reportedly increase straylight [6,7]. Increased forward scattering or straylight can degrade the quality of the retinal image, potentially resulting in decreased visual acuity, impaired visual function, and subjective symptoms such as blurry vision or glare [8].

Decreased contrast sensitivity has been reported in a patient with FED [6]. However, the visual function of affected patients has not been sufficiently examined owing to the epidemiological rarity of these diseases. A comprehensive, quantitative evaluation of visual function in multiple patients that enables statistical analysis has not yet been conducted.

FED and LCAT deficiency are rare diseases with a prevalence of <1/1,000,000 [9,10], making it difficult to prospectively enroll patients in clinical studies to evaluate visual function. We hypothesized that patients with LCAT abnormalities would have generally normal visual acuity but reduced contrast sensitivity and increased forward scattering compared with controls. Accordingly, the aim of the present study was to compare best-corrected visual acuity (BCVA), corneal astigmatism, forward light scattering, and contrast sensitivity between patients with LCAT abnormalities - namely, FED and LCAT deficiency - and healthy controls.

## Materials and methods

Ethics approval and informed consent

The study protocol adhered to the tenets of the Declaration of Helsinki and was approved by the Institutional Review Board of the Miyata Eye Hospital, Miyakonojo, Japan (identifier: CS-280_026). Written informed consent was obtained from all patients.

Study design and patients

This retrospective, comparative case-control study included consecutive patients diagnosed with FLD or FED at Miyata Eye Hospital between January 2018 and December 2024 (LCAT group). Diagnosis was based on characteristic clinical findings on slit-lamp examination, biochemical evidence of absent or decreased serum LCAT activity, and confirmation via genetic analysis [11]. The control group included age- and sex-matched individuals with no history of ophthalmic disease who underwent the same visual function evaluation during the same period. The exclusion criteria for both groups included a history of corneal surgery, the presence of other ocular diseases affecting vision, or visual impairment due to non-corneal causes.

Data collection

Data retrospectively collected from the medical records included patients' background, BCVA, intraocular pressure, central corneal thickness, corneal astigmatism, forward scattering (straylight value), and contrast sensitivity. Central corneal thickness was measured using anterior segment optical coherence tomography (CASIA SS-1000; Tomey Corporation, Nagoya, Japan). BCVA was measured using a standard 5-m Snellen chart and converted to the logarithm of the minimum angle of resolution (logMAR) for analysis. Keratometry was used to measure corneal astigmatism. Forward scattering was assessed using a C-Quant straylight meter (Oculus GmbH, Wetzlar, Germany), and the logarithm of the straylight parameter (log(s)) was recorded, as previously reported [12]. Contrast sensitivity was evaluated using a CSV-1000 chart (Vector Vision, Greenville, OH, USA) under standard photopic conditions at 3, 6, 12, and 18 cycles/degree, with a background photopic luminance of 85 cd/m², according to the manufacturer’s protocol. To plot the curve, the measurement results were converted to log units using a specific table.

Statistical analysis

Normality was tested using the Shapiro-Wilk test. Given the small number of patients and the non-normality of the data, we used the Mann-Whitney U test to compare BCVA, corneal astigmatism, and forward scattering between the LCAT and control groups. Contrast sensitivity data, represented by the area under the log-contrast sensitivity function (AULCSF), were compared between the two groups using the Mann-Whitney U test. AULCSF was calculated from the contrast sensitivity values obtained at spatial frequencies of 3, 6, 12, and 18 cycles/degree.

All statistical analyses were performed using the Bell Curve for Excel (Social Survey Research Information Co., Ltd., Tokyo, Japan). Statistical significance was set at p < 0.05. Data are presented as median (interquartile range), unless otherwise specified.

## Results

Participant characteristics

A total of 16 eyes of eight women were included in the final analysis: eight eyes of four patients in the LCAT group and eight eyes of four controls. In the LCAT group, three patients (six eyes) had FED, and one patient (two eyes) had FLD syndrome. The mean ages of the LCAT and control groups were 64.5 (62.3-66.3) and 63.5 (60-65.3) years (p = 0.54), respectively. The central corneal thickness was significantly greater in the LCAT group than in the control group (p = 0.020). The proportion of patients with phakia (100% vs. 25%) tended to be higher in the LCAT group than in the control group, whereas intraocular pressure tended to be lower in the LCAT group than in the control group (12.0 (9.8-14.3) mmHg vs. 14.5 (13.3-15.0) mmHg); these differences were not significant (Table 1).

**Table 1:** Characteristics of patients with LCAT abnormalities (LCAT group) and control subjects (control group) * indicates p < 0.05 LCAT, lecithin-cholesterol acyltransferase

	LCAT group	Control group	p-value
Age (years)	64.5 (62.3-66.3)	63.5 (60-65.3)	0.54
Female:male	4:0	4:0	1.00
Phakia:pseudophakia (eyes)	8:0	2:6	0.14
Intraocular pressure (mmHg)	12 (9.8-14.3)	14.5 (13.3-15.0)	0.18
Central corneal thickness (μm)	596 (579.5-602.3)	551 (512-575.8)	0.020*

**Figure 1:**
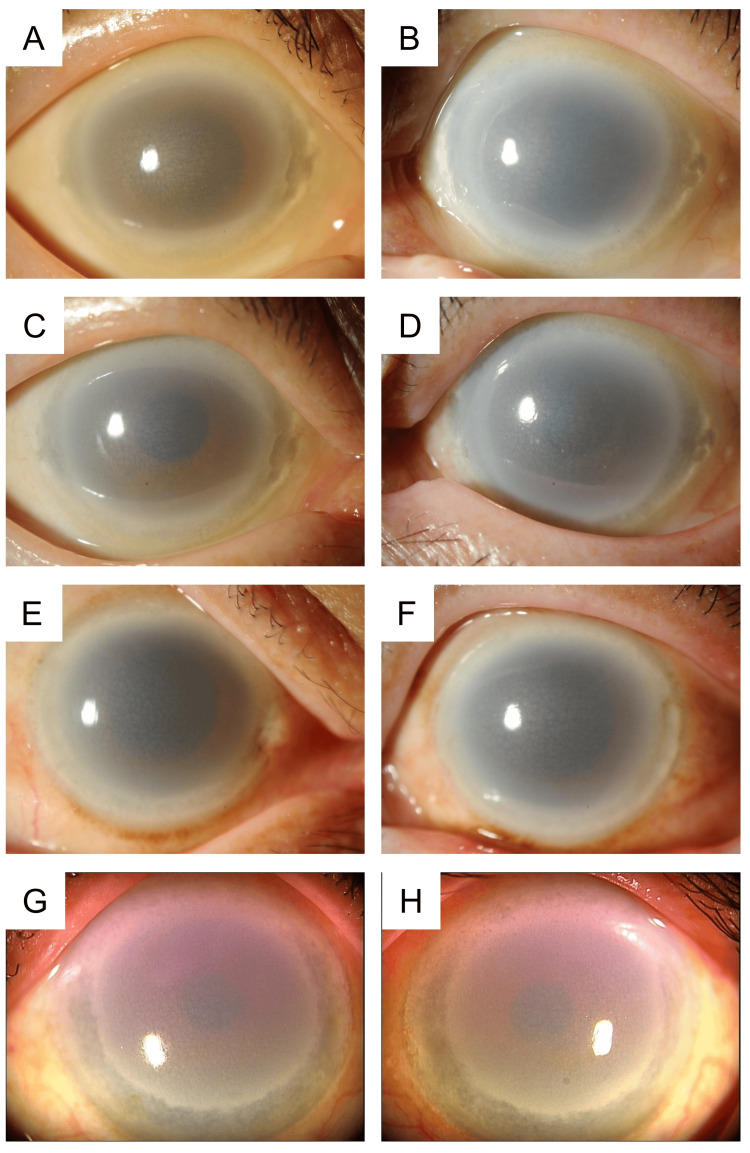
Representative images of the cornea of patients with LCAT abnormalities The corneas of patients with LCAT deficiency and fish-eye disease, who exhibit LCAT abnormalities, are strongly opacified. The density of the opacity is greater at the periphery than at the center. (A, right eye; B, left eye) Images of the anterior segment of a patient with familial LCAT deficiency; (C, right eye; D, left eye) Images of the anterior segment of patient #1 with fish-eye disease; (E, right eye; F, left eye) Images of the anterior segment of patient #2 with fish-eye disease; (G, right eye; H, left eye) Images of the anterior segment of patient #3 with fish-eye disease. LCAT, lecithin-cholesterol acyltransferase

All eyes from the LCAT group are shown in Figure 1. Chief complaints were photophobia in 75% of patients (n = 3) and subjective deterioration of vision in 25% of patients (n = 1).

Visual function

**Figure 2:**
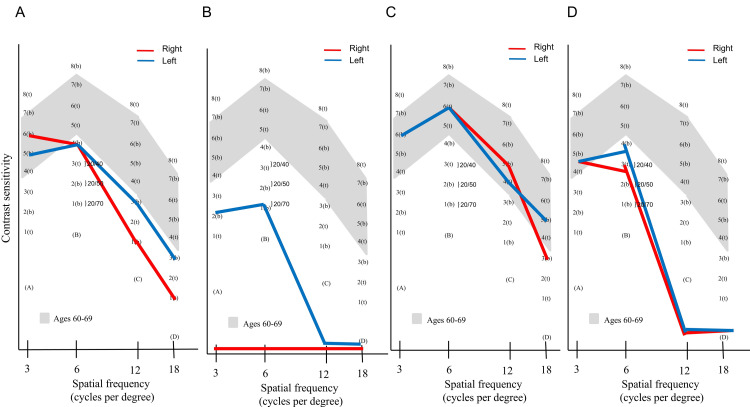
Contrast sensitivity of patients with LCAT abnormality Contrast sensitivity has been evaluated at 3, 6, 12, and 18 cycles/degree (x-axis). Patients with LCAT deficiency and fish-eye disease, who have LCAT abnormalities, show decreased contrast sensitivity at most spatial frequencies. (A) Contrast sensitivity of a patient with familial LCAT deficiency; (B) contrast sensitivity of patient #1 with fish-eye disease; (C) contrast sensitivity of patient #2 with fish-eye disease; (D) contrast sensitivity of patient #3 with fish-eye disease. LCAT, lecithin-cholesterol acyltransferase

Forward scattering was significantly increased in the LCAT group compared with that in the control group (p = 0.007). In the LCAT group, BCVA and corneal astigmatism were 0.00 (-0.02 to 0.06) logMAR and 0.94 (0.64-1.73) diopters (D), respectively, demonstrating no significant difference when compared with values in the control group (-0.18 (-0.18 to -0.04) logMAR and 0.89 (0.27-1.06) D; p = 0.08 and 0.60, respectively). In the LCAT group, the contrast sensitivity at high frequencies decreased from the normal range in seven eyes. Figure 2 shows the contrast sensitivity values in the LCAT group, and Table 2 shows the contrast sensitivity values for each frequency. AULCSF was 1.15 (0.76-1.50) in the LCAT group, significantly lower than that in the control group (1.98 (1.87-2.09); p = 0.017).

**Table 2:** Visual function of patients with LCAT abnormalities (LCAT group) and control subjects (control group) * indicates p < 0.05 LCAT, lecithin-cholesterol acyltransferase; BCVA, best-corrected visual acuity; logMAR, logarithm of the minimum angle of resolution; AULCSF, area under the log-contrast sensitivity function

	LCAT group	Control group	p-value
BCVA (logMAR)	0 (-0.02 to 0.06)	-0.18 (-0.18 to -0.04)	0.08
Corneal astigmatism (diopters)	0.94 (0.64-1.73)	0.89 (0.27-1.06)	0.60
Forward scattering (logarithm of the straylight parameter)	2.15 (2.07-2.32)	0.96 (0.90-1.03)	0.007*
Contrast sensitivity (AULCSF)	1.15 (0.76-1.50)	1.98 (1.87-2.09)	0.017*

## Discussion

In this study, we quantitatively assessed the visual function of patients with LCAT abnormalities, namely, FLD and FED. Our results indicated that, although BCVA and corneal astigmatism were comparable to those of age- and sex-matched controls, forward scattering was considerably increased, and contrast sensitivity was markedly decreased in patients with LCAT abnormalities. Patients with FED and FLD often maintain good BCVA despite the presence of dense corneal opacity [6,13]. This phenomenon might be partly explained by the typical opacity distribution, which tends to be more severe in the peripheral cornea while sparing the central visual axis [14]. Additionally, the value of corneal astigmatism was not large in our study (0.94 (0.64-1.73) D). Irregular astigmatism, which cannot be corrected with spectacles, may not be substantial in these patients, based on these results and the fact that BCVA was good.

Central corneal thickness was markedly greater in the LCAT group in our study. This tendency is consistent with that reported previously [15]. Although this finding suggests the influence of lipid deposits within the corneal stroma, a pathological assessment was not performed in this study. Further investigation is required to assess the correlation of the degree and extent of opacity with visual function. The difference in corneal thickness, although not significant, should also be considered a potential confounder in intraocular pressure measurements. Conversely, forward scattering was substantially higher in the LCAT group. Increased forward scattering is a known consequence of different corneal diseases and contributes to straylight, which degrades retinal image quality [8]. Patients with FED often present with photophobia [11]. The increased forward scattering observed in our LCAT group, likely resulting from lipid deposition in the corneal stroma, might have substantially contributed to subjective symptoms, such as photophobia. Therefore, ophthalmologists should assess and recognize the degree of photophobia and the severity of corneal opacity.

Contrast sensitivity decreased in seven of eight eyes in the LCAT group. A previous case report indicated reduced contrast sensitivity in a patient with FED [6], which is consistent with our findings. Notably, 75% of the patients in our LCAT group were conscious of photophobia, and 25% complained of deterioration of vision despite having a normal BCVA. This finding underscores the fact that alterations in contrast sensitivity may markedly influence subjective visual performance, even when standard acuity measurements appear normal. Although the sample size was small and the preliminary findings may not be generalizable to all cases, the effect sizes for the significant results (forward scattering and contrast sensitivity) were substantial. All cases included in the current LCAT group were women, and, while age-related hormonal abnormalities play a significant role in corneal thinning, changes in female hormones do not affect contrast sensitivity [16].

The impact of systemic diseases on visual function has recently garnered attention. Koh et al. reported visual function in patients with cornea verticillata associated with Fabry disease and emphasized that conventional visual acuity alone does not fully capture visual function [17]. The analysis of contrast sensitivity across various spatial frequencies provides further insights. Our findings (Table 2 and Figure 2) indicate a decrease in contrast sensitivity, predominantly at intermediate and high spatial frequencies, in the LCAT group. The observed pattern of decreased contrast sensitivity, especially at intermediate and high frequencies, may be attributed to the diffuse nature of the light scattered by corneal opacities, even if the central cornea retains relative clarity. Future studies comparing these results with those of patients with normal contrast sensitivity could further elucidate the specific impact of LCAT-related opacities.

The AULCSF serves as a comprehensive index of contrast sensitivity across different spatial frequencies and is a valuable measure of overall visual function [18]. Reduced AULCSF, as observed in our LCAT group compared to that in the controls, has been reported in other corneal diseases that cause opacity and often correlates well with the subjective visual complaints of patients, even when BCVA remains relatively normal [19-24]. The AULCSF can sensitively detect subtle alterations in visual function that are not captured by standard BCVA measurements, making it a widely used tool. The substantial reduction in AULCSF in our study is consistent with the data calculated from the contrast sensitivity test in a previous case report by Kanai et al. [6], which also documented decreased contrast sensitivity in a patient with FED.

This study has some limitations. First, the number of patients included in this study was small because LCAT abnormalities are rare. The small sample size may have resulted in a larger standard error in the statistical analyses. Second, owing to the retrospective nature of the study, six eyes of three patients in the control group were pseudophakic, in contrast to all phakic eyes in the LCAT group. Van den Berg et al. suggested that lens extraction can reverse age-related increases in straylight, independent of visual acuity [25]. The lens condition must be unified because pseudophakia has been reported to influence contrast sensitivity [20]. The presence of individuals with pseudophakia in the control group may have resulted in better contrast sensitivity, potentially exaggerating the difference between groups. Further prospective studies, including patients with more closely matched characteristics - especially lens status - are required to compare the groups more accurately.

## Conclusions

In summary, patients with LCAT abnormalities, namely FLD and FED, showed markedly elevated forward scattering and reduced contrast sensitivity compared with the control eyes, despite maintaining normal BCVA. These results underscore the criticality of assessing parameters beyond BCVA, such as forward scattering and contrast sensitivity, to fully understand visual functional impairment in these patients.
